# Surface Plasmon Resonance Biosensor Method for Palytoxin Detection Based on Na^+^,K^+^-ATPase Affinity

**DOI:** 10.3390/toxins6010096

**Published:** 2013-12-27

**Authors:** Amparo Alfonso, María-José Pazos, Andrea Fernández-Araujo, Araceli Tobio, Carmen Alfonso, Mercedes R. Vieytes, Luis M. Botana

**Affiliations:** 1 Department of Pharmacology, Veterinary School, University of Santiago de Compostela, 27002 Lugo, Spain; E-Mails: amparo.alfonso@usc.es (A.A.); andrea.fernandez.araujo@rai.usc.es (A.F.-A.); araceli.tobio@rai.usc.es (A.T.); 2 Confocal and Electronic Microscopy Unit, RIAIDT, University of Santiago de Compostela, 27002 Lugo, Spain; E-Mail: m.pazos@usc.es (M.-J.P.); 3 Department of Physiology, Veterinary School, University of Santiago de Compostela, 27002 Lugo, Spain; E-Mail: mmercedes.rodriguez@usc.es; 4 Cifga Laboratory, Santo Domingo Square, 20, 5ª, 27001 Lugo, Spain; E-Mail: mcalfonso@cifga.es

**Keywords:** palytoxin, Na^+^,K^+^-ATPase, surface plasmon resonance biosensor, *Ostreopsis siamensis*

## Abstract

Palytoxin (PLTX), produced by dinoflagellates from the genus *Ostreopsis* was first discovered, isolated, and purified from zoanthids belonging to the genus *Palythoa*. The detection of this toxin in contaminated shellfish is essential for human health preservation. A broad range of studies indicate that mammalian Na^+^,K^+^-ATPase is a high affinity cellular receptor for PLTX. The toxin converts the pump into an open channel that stimulates sodium influx and potassium efflux. In this work we develop a detection method for PLTX based on its binding to the Na^+^,K^+^-ATPase. The method was developed by using the phenomenon of surface plasmon resonance (SPR) to monitor biomolecular reactions. This technique does not require any labeling of components. The interaction of PLTX over immobilized Na^+^,K^+^-ATPase is quantified by injecting different concentrations of toxin in the biosensor and checking the binding rate constant (*k*_obs_). From the representation of *k*_obs_* versus* PLTX concentration, the kinetic equilibrium dissociation constant (*K*_D_) for the PLTX-Na^+^,K^+^-ATPase association can be calculated. The value of this constant is *K*_D_ = 6.38 × 10^−7^ ± 6.67 × 10^−8^ M PLTX. In this way the PLTX-Na^+^,K^+^-ATPase association was used as a suitable method for determination of the toxin concentration in a sample. This method represents a new and useful approach to easily detect the presence of PLTX-like compounds in marine products using the mechanism of action of these toxins and in this way reduce the use of other more expensive and animal based methods.

## 1. Introduction

Palytoxin (PLTX), isolated from the marine soft coral (genus *Palythoa*), is one of the most poisonous non-protein substances known to date. It is common in tropical and subtropical waters and may accumulate at very high levels in fish and crabs [1]. Animals may incorporate PLTX by filtering and therefore entering the toxin in the food chain [2]. PLTX is extremely potent through intravenous, intraperitoneal, and intratracheal exposure, and less potent by direct intragastric exposure [3]. Due to co-occurrence with other seafood toxins, such as ciguatoxins, saxitoxins, and tetrodotoxin, it has been difficult to assess the true risk of PLTX poisoning through seafood consumption in humans [4]. Toxin quantification and identification in seafood has relied on different methodologies, mainly LC-MS, mouse bioassay, hemolysis neutralization assay and ELISA assays [5,6,7,8,9,10,11]. The large spatial expansion of this toxin has led to intensification of research towards optimizations of methods for determination of PLTX presence and toxicity. This toxin is a large, very complex molecule with both lipophilic and hydrophilic areas, and has the longest chain of continuous carbon atoms known to exit in a natural product. Several molecules related with PLTX have been described: palytoxin-b, homopalytoxin, bishomopalytoxin, neopalytoxin, deoxypalytoxin, 42-hydroxypalytoxin, ostreocin-d, ovatoxin-a, -b, -c,-d, -e and -f and mascarenotoxin-a, -b and -c. However only the chemical structures of PLTX, ostreocin-d, ovatoxin-a and 42-hydroxypalytoxin have been characterized [12,13,14,15,16,17,18].

Several studies indicates that PLTX binds to the Na^+^,K^+^-ATPase in the plasma membrane of animal cells and opens a cation pathway through the pump [19,20,21]. Therefore, the Na^+^,K^+^-ATPase has been proposed as for the toxin receptor. PLTX has an effect on primary neuronal cultures of cerebellar granule cells (CGC), leading to a large increase in the cytosolic calcium concentration and to a large intracellular acidification of these neurons [22,23]. Several symptoms like scratching, jumping, paralysis of hind limbs, respiratory distress, cyanosis also brings nausea, tiredness, diarrhoea and vomiting followed by dizziness, in animals have been descrived [24]. In humans PLTX poisoning is called palytoxicosis or clupeotoxicosis [25]. This process is associated also to intestinal symptoms, muscle spasms, breathing difficulties followed in some cases by dead in humans have been descrived as consequence of contaminated food consoptium [24,26,27]. 

The use of optical biosensors to study molecular interactions is a well accepted method. This technology has been used to measure in real time the binding kinetics between a macromolecule in solution and a receptor immobilized. In this way, fundamental information over biospecific interactions can be obtained. Many approaches have been done to develop marine toxin and food contaminants detection methods employing the biosensors technology by using either antibodies or toxin receptors with high success [28,29,30,31,32,33].

In this paper, we used a rapid surface plasmon resonance (SPR) biosensor assay to study the interaction of PLTX and ouabain, as control, with immobilized Na^+^,K^+^-ATPase from dog kidney and to develop a new method to detect the toxin in shellfish. The technique does not require any labeling of the interacting components and the interactions are measured in real time.

## 2. Results and Discussion

We have shown that ouabain interacts with a sensor surface-attached Na^+^,K^+^-ATPase, however no interaction between the ATPase and PLTX was observed [23]. However, the technology used, an Iasys Affinity Sensor, and the chemical reactions needed to attach the protein prevented to show any interaction. In the present paper, we used another biosensor, Biacore X SPR, and different chemical approaches in order to study and to measure the binding between PLTX and the Na^+^,K^+^-ATPase. The Na^+^,K^+^-ATPase was used as the ligand attached to the sensor surface and PLTX in solution was used as the ligate.

**Figure 1:**
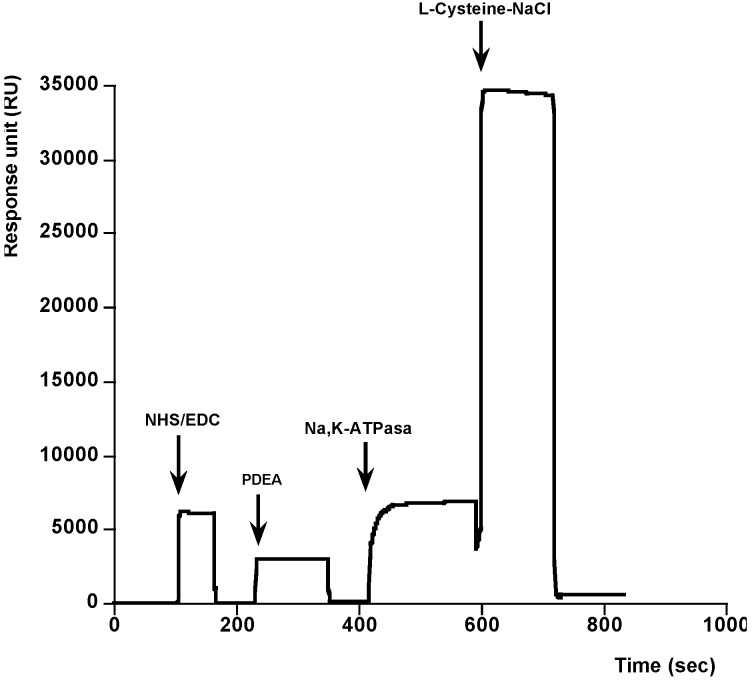
Sensor surface activation and Na^+^,K^+^-ATPase immobilization. Activation: first arrow shows the addition of EDC/NHS to activate the CM5 dextran. Derivatisation: the second arrow shows the addition of 80 mM PDEA to the surface to derivatizate. Immobilization: the third arrow indicates the addition of 100 µg mL^−1^ Na^+^,K^+^-ATPase from dog kidney. Blocking: the remaining activated sites were blocked with cysteine/NaCl, fourth arrow.

Initially the Na^+^,K^+^-ATPase was immobilized over a CM5 sensor chip previously activated. Amine coupling is the most generally applicable coupling chemistry because most macromolecules contain many groups whose can participate in the amine coupling reaction, and therefore the immobilization is usually easy [34]. However, as it was shown this kind of immobilization prevented PLTX binding [23]. In this sense, there are situations where other coupling methods may be preferable because ligands have active site that include particularly reactive amino groups that may lose biological activity on immobilization. In these cases, a coupling by thiol-disulphide exchange, by introducing an active disulphide on the sensor chip surface and exchanging with intrinsic thiol groups in the ligand is used [35]. The efficiency of thiol coupling is very high, and the conditions for immobilization are often less critical than with amine coupling. This was the strategy followed in the present paper. As Figure 1 shows, 100 µg mL^−1^ of Na^+^,K^+^-ATPase from dog kidney dissolved in sodium acetate was added, 410 s, to the sensor chip performed by introducing an active disulphide on the sensor chip surface. In these conditions, a typical covalent binding curve was obtained. Then, the surface was washed with HBS buffer flow and no fall in the signal was observed, indicating that Na^+^,K^+^-ATPase was strongly immobilized onto the surface of the sensor chip. Finally cysteine/NaCl was injected (600 s to 740 s) to deactivate reactive disulphides excess and remove non-covalently bound protein.

**Figure 2:**
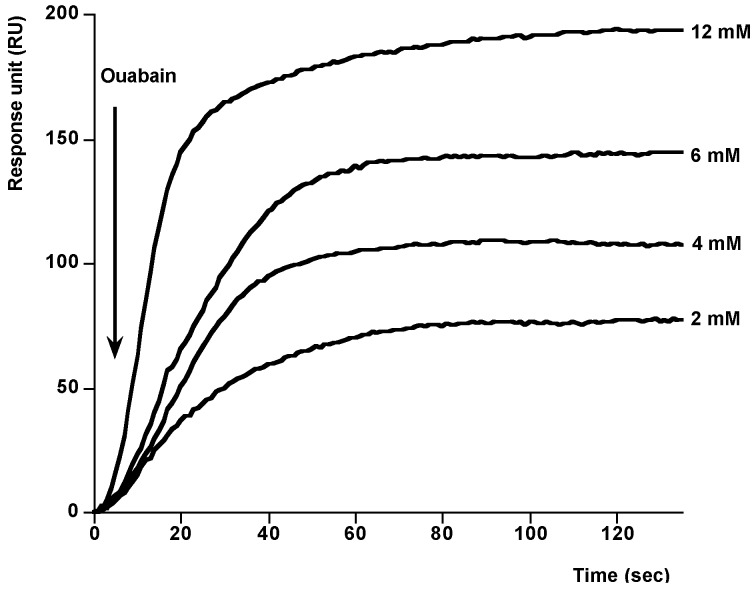
Ouabain-Na^+^,K^+^-ATPase association. Association curves after addition of different amounts of ouabain to immobilized Na^+^,K^+^-ATPase. Different Ouabain concentrations were injected using HBS-EP as running buffer and a flow rate of 10 µg mL^−1^. The association curves were obtained after subtraction of their respective solvent control. Representative of 4 experiments.

**Figure 3:**
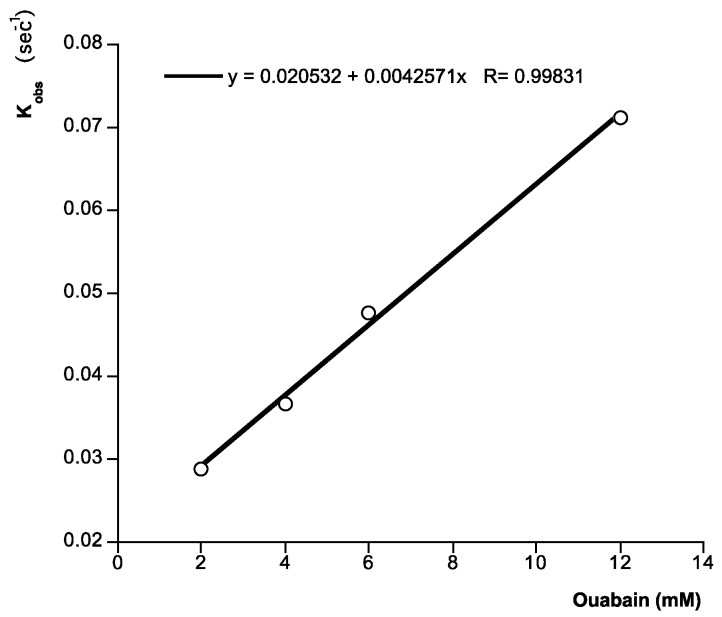
Analysis of ligand binding. Kinetic plot of apparent association rate constant *K*_obs_ (s^−1^) obtained from plot in Figure 2 (calculated by the BiaEvaluation software) *versus* ouabain concentration. Representative of 4 experiments.

Next, to check the activity of immobilized Na^+^,K^+^-ATPase, different concentrations of ouabain were added at 25 °C, by using HBS-EP as running buffer at flow rate of 10 µL min^−1^. In this case, typical association curves were observed. As Figure 2 shows, the responses after 120 s ouabain addition follow a typical association curve profile. In the presence of 2 mM ouabain the signal is 87.04 RU, while in the presence of 12 mM ouabain the response reaches 178.99 RU. The individual binding curves from Figure 2 were analyzed to determine the kinetic constants of ouabain-Na^+^,K^+^-ATPase binding, namely, the observed rate constant (*k*_obs_), the association rate constant (*K*_ass_), the dissociation rate constant (*K*_diss_), and the kinetic equilibrium dissociation constant (*K*_D_). At equilibrium, by definition, *K*_diss_/*K*_ass_ = *K*_D_. The pseudo-first-order association rate constant *k*_obs_ (s^−1^) was determined for each ouabain concentration by using the 1:1 Langmuir association model of the BiaEvaluation software (BiaCore, Uppsala, Sweden). Figure 3 shows a representation of each *K*_obs_ against the corresponding concentration of ouabain (representative of one experiment). This plot follows a linear correlation coefficient, *r* = 0.99. From the equation of this representation, *K*_ass_, M^−1^ s^−1^, gradient of the plot, and *K*_diss_, s^−1^, intercept of the plot were obtained. Within these two values, the kinetic equilibrium dissociation constant *K*_D_ (*Y*-intercept/slope) for the ouabain-Na^+^,K^+^-ATPase binding was obtained. The value of this constant was 4.2 × 10^−3^ ± 8.4 × 10^−4^ M ouabain (average of at least three experiments). This value is similar to the value obtained in our laboratory to the same experiment in direct binding assays with a resonant mirror biosensor [23].

**Figure 4:**
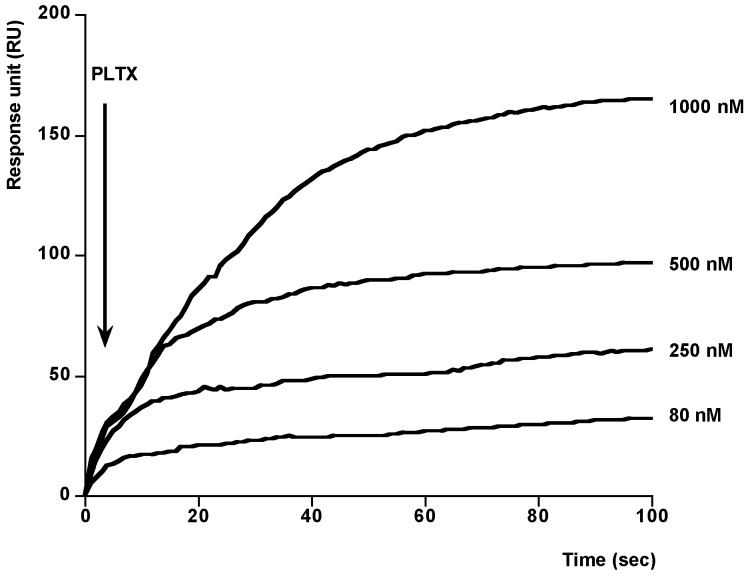
PLTX-Na^+^,K^+^-ATPase association. Association curves after addition of different amounts of PLTX to immobilized Na^+^,K^+^-ATPase. Different PLTX concentrations were injected over the CM5 chip using HBS-EP as running buffer and a flow rate of 10 µL min^−1^. The association curves were obtained after subtraction of their respective solvent control. Representative of 4 experiments.

**Figure 5:**
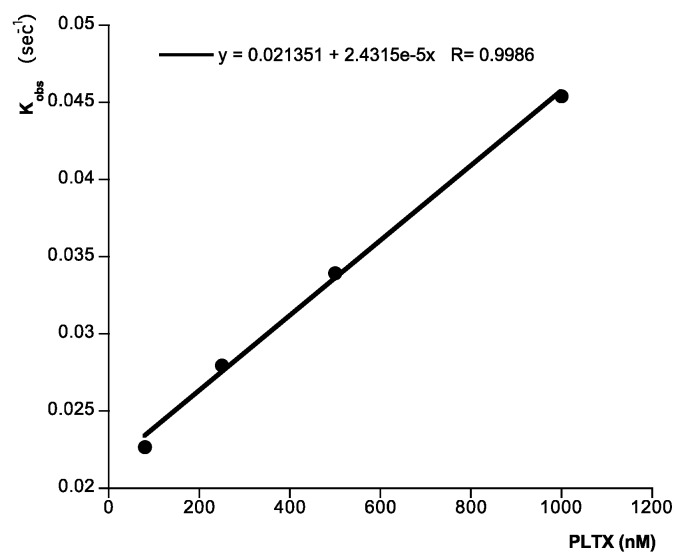
Analysis of ligand binding. Kinetic plot of apparent association rate constant *K*_obs_ (s^−1^) obtained from plot in Figure 4 (calculated by the BiaEvaluation software) *versus* ouabain concentration. Representative of 4 experiments.

**Figure 6:**
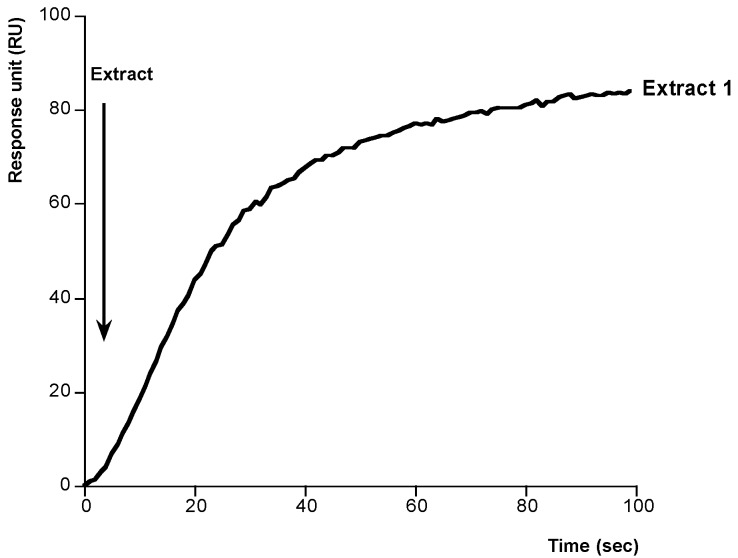
Determination of PLTX concentration in *Ostreopsis siamensis* extract 1. Association curve after addition of extract 1 dissolved in methanol-water to immobilized Na^+^,K^+^-ATPase. The *K*_obs_ (s^−1^) value was calculated from this curve and transformed in toxin concentration using the equation obtained from plot in Figure 5.

**Figure 7:**
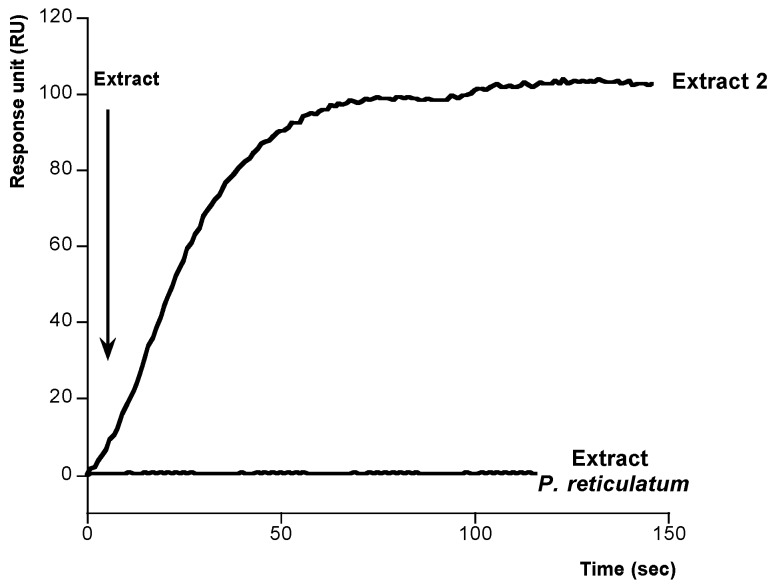
Determination of PLTX concentration in *Ostreopsis siamensis* extract 2 and *Prorocentrum reticulatum*. Association curves after addition of extracts dissolved in methanol-water to immobilized Na^+^,K^+^-ATPase. From extract 2 curve the *K*_obs_ (s^−1^) value was calculated and transformed in toxin concentration using the equation obtained from plot in Figure 5.

After setting up these conditions we developed a method to study the binding PLTX-Na^+^,K^+^-ATPase. In this case, different concentrations of PLTX were dissolved in methanol-water (1:1) and added onto the immobilized Na^+^,K^+^-ATPase. As Figure 4 shows, typical association curves were obtained when PLTX was added. After 100 s, responses from 30 to 170 RU, depending on the PLTX concentration, were obtained. The PLTX-Na^+^,K^+^-ATPase interaction follows a pseudo-first-order kinetics where *k*_obs_ can be calculated. The value of this constant is increasing with PLTX concentration. When *K*_obs_ was represented against the corresponding concentration of PLTX a linear regression with a correlation coefficient of *r* = 0.9986 was obtained, Figure 5. From this representation the kinetic equilibrium constant for the PLTX-Na^+^,K^+^-ATPase binding was obtained *K*_D_ = 6.38 × 10^−7^ ± 6.67 × 10^−8^ M PLTX. This value is in the range of *K*_D_ for unions between active biological species (10^−11^ and 10^−4^ M) [36]. After these results, the method was used to detect PLTX in contaminated samples. The amount of toxin in two *Ostreopsis siamesis* extracts and one *Prorocentrum reticulatum* (*P. reticulatum*) culture, as negative control, was quantified and the results compared with the amount obtained by fluoresce polarization (FP) other PLTX detection assay recently described [5]. *Ostreopsis* spp. have been described as PLTX-like compounds producers [37]. As Figure 6 and Figure 7 show, the typical association binding curve were obtained from Na^+^,K^+^-ATPase-PLTX union when *Ostreopsis* spp. cultures were added. However, as Figure 7 shows *P. reticulatum* culture did not induce any signal increase. The *k*_obs_ values calculated from these curves can be transformed in PLTX concentration using the equation obtained from the calibration curve (Figure 5). In this case, the PLTX concentrations obtained with the biosensor were 56.2.8 ± 8.83 µM and 670 ± 31.6 µM and with the FP assay 68.8 ± 9.8 µM and 761.1 ± 4.5 µM (Table 1). Therefore this method is reliable, the results obtained are comparable with FP method, and has a high degree of repeatability. The biosensor method avoids the ethical problems of the official mousse bioassay and the false-positive and false-negative results. In addition, the biosensor method here proposed is a fast and accurate method where only PLTX or like-PLTXs compounds are detected [7,24,38]. The limit of detection (LOD) for SPR method is 3.73 pg PLTX and the limit of quantification (LOQ) is 11.2 pg PLTX. These limits are comparable with other biological PLTX detection methods as the fluorescent polarization assay [5,7], the cytolytic assay [8] or the sandwich ELISA assay [11], but maybe not as good as the hemolysis or the EILISA assays [9,10]. In addition, a toxic dose in humans would be between 2.3 and 31.5 µg [39], which is higher than the lower limit of detection of this assay.

**Table 1:** Concentration of PLTX in *Ostreopsis siamensis* and *P. reticulatum* extracts. PLTX concentrations were obtained by using the biosensor method and the fluoresce polarization method [5].

	PLTX (µM) Biosensor assay	PLTX (µM) FP assay
Extract 1	56.2.8 ± 8.83	68.8 ± 9.8
Extract 2	670 ± 31.6	761.1 ± 4.5
*P. reticulatum*	-	-

## 3. Materials and Methods

### 3.1. Instrumentation

A Biacore X SPR biosensor with Control Software and BIAevaluation software version 3.0 were purchased from Biacore (GE Healthecare, Uppsala, Sweden).

### 3.2. Chemicals

PLTX was obtained from Wako Pure Chemical Industries (Neuss, Germany). CM5 sensor chips, HBS-EP buffer (pH 7.4, 0.01 M HEPES, 0.15 M NaCl, 3 mM EDTA, 0.005% polysorbate), amine coupling kit (1-ethyl-3-(3-dimetylaminopropyl) carbodiimide hidrochloride (EDC) and *N*-hydroxysuccinimide (NHS) and 2,2-pyridinyldithio-ethaneamine (PDEA) were supplied by Biacore AB (Uppsala, Sweden).

l-Cysteine, Boric Acid, Sodium Formate, Sodium Hydroxide were purchased from Sigma Chemical Co. (Madrid, Spain). All buffered solutions were degassed and filtered through a Millex^®^ GP 0.22 µm pore size filter from Millipore Corporation (Bedford, MA, USA).

Ouabain and Na^+^,K^+^-ATPase isolated and purified from dog kidney were purchased from Sigma (St Louis, MO, USA).

### 3.3. Methods

#### 3.3.1. Surface Activation and Ligand Inmobilization

Sensor surface activation and ligand immobilization were performed using HBS-EP as running buffer at a flow rate of 5 µL min^−1^ and 25 °C. The CM5 dextran matrix chip was activated using an amine coupling kit. Following manufacture instructions, a mixture (1:1, *v*/*v*) of EDC and NHS was applied for 2 min over the sensor chip. After removal excess solution, 80 mM PDEA dissolved in 0.1 M borate buffer at pH 8.5 was injected for 4 min to derivatize the sensor chip. Next the ligand, 100 µg/mL of Na^+^,K^+^-ATPase from dog kidney dissolved in sodium acetate 10 mM at pH 4.5, was added. Finally cysteine/NaCl was injected to deactivate excess reactive disulphides, to remove non-covalently bound protein and therefore to block remaining activated sites.

#### 3.3.2. Binding of PLTX or Ouabain to Immobilized Na^+^,K^+^-ATPase

Several Ouabain concentrations diluted in water were added over sensor chip to bind with the immobilized Na^+^,K^+^-ATPase. The duration of the sample injection was 2 min at 10 µL min^−1^ flow rate. Next, dissociation of bounded ouabain in HBS buffer flow was studied. The bounded alkaloid to the chip surface was removed before the next injection by adding 1 M glycine-HCl at pH 2.5 for 1 min.

A range of PLTX concentrations were diluted in methanol-water and added over sensor chip to study the binding between PLTX and Na^+^,K^+^-ATPase immobilized. The dissociation was done as before.

Control solutions (with methanol/water) were injected in the second flow cell in the same way and subsequently subtracted from ligate response signal.

Since toxin dissociates almost completely, we used the association phase to quantify the toxin-Na^+^,K^+^-ATPase interaction.

### 3.4. Dinoflagellate Extracts

Cultures of *Ostreopsis siamensis*, were grown (2 L glass conical flask) in seawater enriched with L1 medium and GSe medium (for *Prorocentrum reticulatum* culture) without silicates. The salinity, 33‰, was adjusted at these proportions by the addition of freshwater to seawater and removing chlorine by aeration. The experiments were carried out at 18–19 °C and the cultures were subjected to photoperiod with day-light lamps on a 16:8 h light-dark photo-cycle. Cells were shaken manually twice a day. The strain growth was divided in steps by progressively increasing the culture volume. The dinoflagellates were kept under these conditions until the beginning of the stationary phase which was reached 30 days after inoculation. The cells were counted using an Utermöhl camera. The whole culture was filtered, the cells were resuspended in methanol/water (1:1), sonicated to their completely homogenization and stored frozen until use.

### 3.5 Statistical Analysis

All experiments were carried out at least three times, each by duplicate and data were normalized. Results were analyzed using the Studentʼs *t*-test for unpaired data. A probability level of 0.05 or smaller was used for statistical significance. Results were expressed as the means ± standard error or the mean (SEM).

## 4. Conclusion

The use of SPR chip surfaces is a new, easy and useful tool for detection of several marine toxins. By using a SPR chip and an amine coupling no binding between PLTX and Na^+^,K^+^-ATPase was previously observed suggesting that additional requirements could be necessary to study the interaction between both molecules. The lack of binding was probably due to some steric impediment of the larger molecule, PLTX MW 2680, or to the blockade of some ligate group into the surface chip essential to the binding [23]. In the present paper, by using a different coupling, thiol-disulphide exchange, the interaction between PLTX and the Na^+^,K^+^-ATPase can be demonstrated. This interaction has value of *K*_D_ = 6.38 × 10^−7^ ± 6.67 × 10^−8^ M PLTX, indicating a high affinity between these two molecules. With this design a detection method for the toxin is developed. The method reported supports the value of biosensor assays as screening methods that would help to reduce the use of other more expensive and ethically questionable methods without putting at risk human health protection.
